# Penile Mondor's disease: a case report

**DOI:** 10.1186/1757-1626-1-411

**Published:** 2008-12-22

**Authors:** Panagiotis Kartsaklis, Charalampos Konstantinidis, Charalampos Thomas, Maria Tsimara, Sotirios Andreadakis, Aristomenis Gekas

**Affiliations:** 1 General Hospital of Patras "O Aghios Andreas", Tsertidou 1, 263 35, Patras, Greece

## Abstract

**Introduction:**

Thrombosis or thromboflebitis of the superficial dorsal vein of the penis, known as penile Mondor's disease was first described by Braun-Falco in 1955.

**Case presentation:**

A physically healthy 32-year-old man proceed in our unit suffered from a painful swelling, on the dorsal aspect of his penis, being more painful during erections. Ultrasonography examination revealed a non-compressible portion of superficial dorsal vein as well as the lack of venous flow signals in Doppler ultrasonography and the patient was treated conservatively.

**Conclusion:**

Penile Mondor's disease is a rare clinical entity that every urologist should be able to recognize. Although it is a benign condition and usually self-limited, patients proceed to specialist with considerable psychological stress.

## Introduction

Superficial vein thrombosis was first described by Mondor in 1939 occurred subcutaneous veins of the anterolateral thoraco-abdominal wall[1]. The most commonly affected vessels are the thoracoepigastric, lateral throracic, and superior epigastric veins, therefore clinical findings have been usually founded in the brest, axilla, groin and posterior cervical region[2]. In 1955, Braun-Falco described penile participation and in 1958 was described by Helm and Hodge an isolated superficial penile vein thrombosis[3]. Herein, we describe the manifestation of the disease in a 32 years old man.

## Case presentation

A 32-year-old man proceed in our unit suffered from painful erections. Our patient noticed over 10 days the appearance of a painful cord on the dorsal aspect of his penis, near the penis root, being more painful during erections. He denied any history of recent trauma, vigorous sexual activity or use of constrictor devices. In addition, he reported that he did not have fever or symptoms from the lower urinary tract.

**Figure 1:**
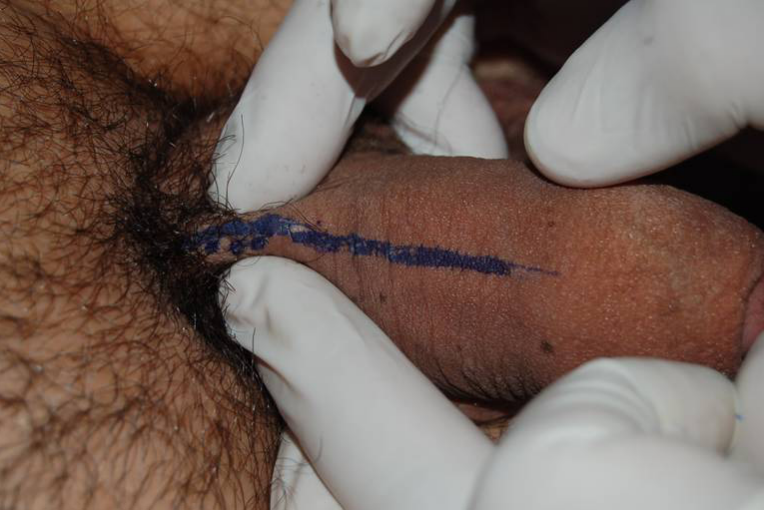
**Superficial trombosed dorsal vein**.

**Figure 2:**
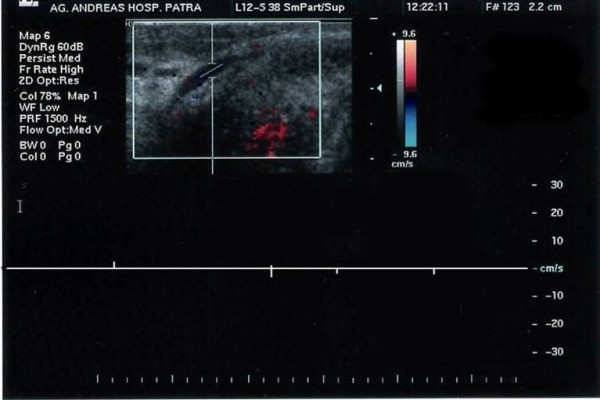
**There is no compressibility of the vein or blood flow indicating thrombosis**.

Physical examination revealed a stressed out but physically healthy man with a dorsal cord-like swelling, extending from the pubic symphysis to mid-shaft of his penis, painful during palpation (Figure 1). Genitourinary examination was normal and the standard laboratory tests (blood and urine) were without pathologic findings. The patient underwent ultrasonography examination which revealed a non-compressible portion of superficial dorsal vein as well as the lack of venous flow signals in Doppler ultrasonography (Figure 2).

The diagnosis indicated superficial dorsal vein trombophlebitis and conservative treatment was prescribed. Treatment included local dressing with heparin ointment and oral treatment with non-steroidal anti-inflammatory (8 mg Lornoxicam), 325 mg of acid salicylic and 500 mg Cefuroxime. The patient was advised to abstain from sexual activity until the symptoms resolved and was scheduled for follow-up in a week, a month and two months.

One week after his first visit patient reported a significant decrease of the pain but the swelling was palpable. Antibiotic discontinued at that time and he was instructed to continue his treatment with non-steroidal anti-inflammatory and acid salicylic.

**Figure 3:**
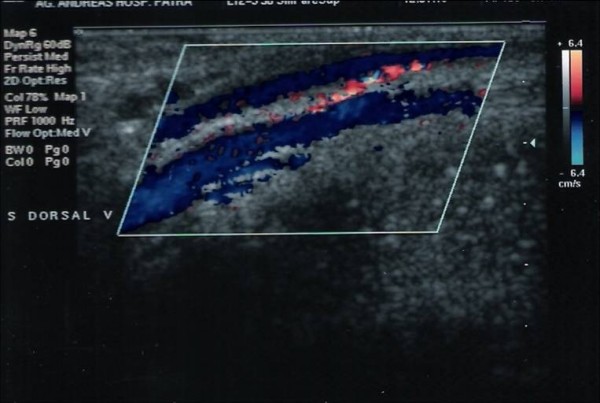
**Normal blood flow is present 40 days after disease's onset**.

At his second medical examination, after a month, about 40 days after the first episode of pain, he had no symptom and the physical examination did not revealed anything pathologic. The patient mentioned the total absence of any symptom approximately 10 days ago. In addition, a Doppler ultrasonography of the superficial dorsal penile vein demonstrated the presence of normal blood flow (Figure 3). Oral treatment stopped and the last visit was considered unnecessary.

## Discussion

In 1939 a French surgeon, Henry Mondor, was the first who described in detail the phlebitis of the chest wall in women. This condition is three times more common in women than in men[4]. Penile Mondor's disease was described in 50's from Braun-Falco and by Helm and Hodge and the incidence recently has been estimated in 1,39%[5].

The aetiology of the disease is poorly understood but vigorous sexual activity, trauma, surgery of the pelvis or external genitalia, prolonged abstinence, contact with menstrual blood which possibly acting as an irritant and tumors in the small pelvis are some of the predisposing factors[6]. Furthermore, penile Mondor's disease was reported a) after long-haul flight[6], b) in a 47-year-old black man with sickle cell trait[1], c) associated with bladder and prostate cancer[1], d) as an unusual manifestation of metastatic pancreatic adenocarcinoma[1] and e) as idiopathic conditions. Regarding to idiopathic conditions, deficiency of protein S, an anti-trombus plasma protein, is considered as a risk factor[3]. The pathogenesis of thrombophlebitis can be attributed to Virchow's triad: damage to vessel wall integrity, changes in blood flow, and changes in the blood components themselves[6,7].

The diagnosis of the disease can be established from the anamnesis and the physical examination. Doppler ultrasonography is a useful and sufficient diagnostic tool for the assessment of venous trombosis. Sclerosing lymphangitis and Peyronie's disease being painful and presenting penile fibrotic lesions, should be considered in the differential diagnosis[7].

About penile Mondor's disease treatment have been proposed several methods convergent to the recommendation of abstinence from sexual intercourse until the resolution of the symptoms. Anticoagulation with heparin or aspirin is recommended. The use of antibiotics is prophylactic or can be necessary in case of cellulites. The double role of non-steroidal anti-inflammatory drugs, provided pain relief and diminished inflammatory reaction, made them useful on specialist's armament[7]. In cases of insisting pain, local injected anesthetics have been used. At last, in cases with no resolution, despite conservative treatment, thrombus excision was treatment of election.

In conclusion, disease has proven to be self-limited, benign and its natural course is three weeks to six months. Although Mondor's disease is often a disease with spontaneous resolution and the treatment is considered palliative, we believe that oral therapy is necessary, accelerating the evolution.

## Consent

Written informed consent was obtained from the patient for publication of this case report and accompanying images. A copy of the written consent is available for review by the Editor-in-Chief of this journal.

## Competing interests

The authors declare that they have no competing interests.

## Authors' contributions

KP was involved in drafting the manuscript and revising it critically for content. KP and TC were the treating urologists, involved in the diagnostic work-up and management of the patient and were involved in revising the draft critically for content. KC and TM performed the ultrasonography examination pre and post-treatment, prepared the images and the relevant legends and were involved in revising the draft critically for content. AS reviewed the international literature and was involved in revising the draft critically for content GA is the consultant of the Section of Andrology and was also responsible for the format and revisions of the manuscript. All authors read and approved the final manuscript.
